# Supplementary material for: Causal Relationship between Aging and Anorexia Nervosa: A White-Matter-Microstructure-Mediated Mendelian Randomization Analysis
Source: Biomedicines. 2024 Aug 16;12(8):1874. doi: 10.3390/biomedicines12081874 (PMC11351342; doi:10.3390/biomedicines12081874)
Supplement: Supplementary file 1 [file biomedicines-12-01874-s001.zip › Supplementary Figure/Figure S4/Singe SNP plot of MR analysis for TL to ubm-b-2094.pdf]

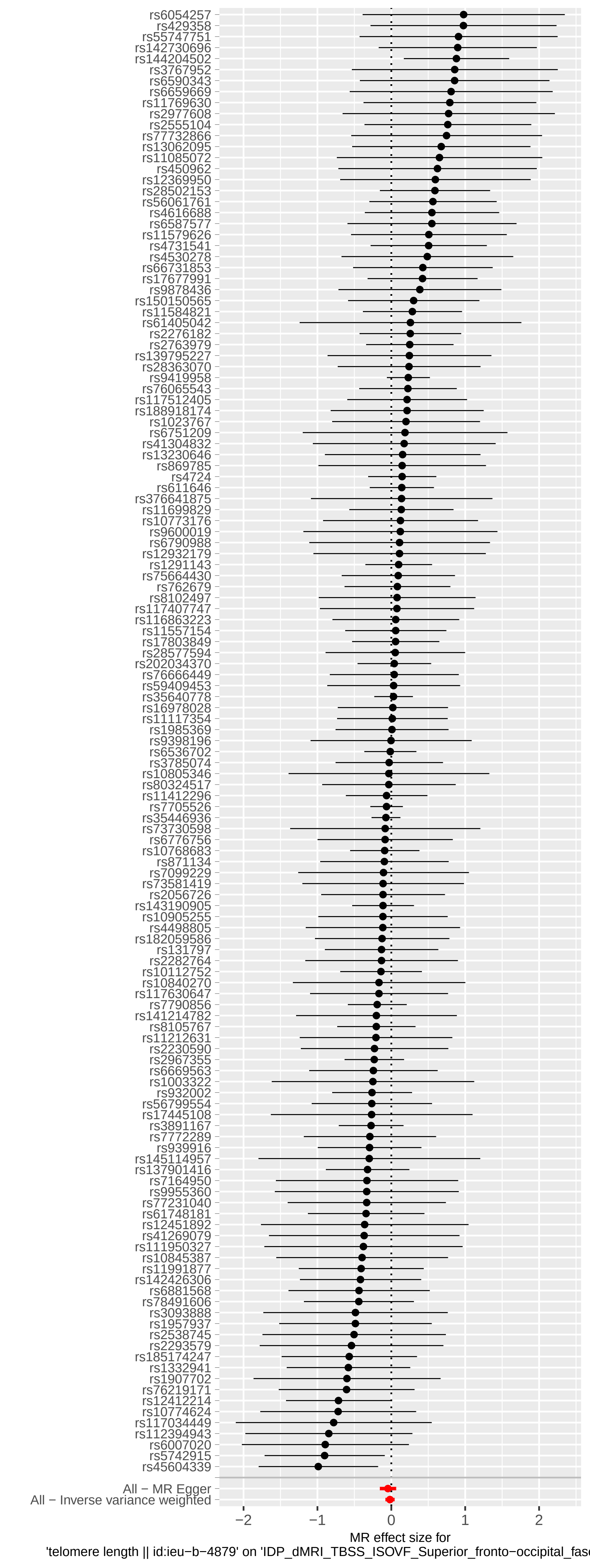
rs6054257
rs429358
rs55747751
rs142730696
rs144204502
rs3767952
rs6590343
rs6659669
rs11769630
rs2977608
rs2555104
rs77732866
rs13062095
rs11085072
rs450962
rs12369950
rs28502153
rs56061761
rs4616688
rs6587577
rs11579626
rs4731541
rs4530278
rs66731853
rs17677991
rs9878436
rs150150565
rs11584821
rs61405042
rs2276182
rs2763979
rs139795227
rs28363070
rs9419958
rs76065543
rs117512405
rs188918174
rs1023767
rs6751209
rs41304832
rs13230646
rs869785
rs4724
rs611646
rs376641875
rs11699829
rs10773176
rs9600019
rs6790988
rs12932179
rs1291143
rs75664430
rs762679
rs8102497
rs117407747
rs116863223
rs11557154
rs17803849
rs28577594
rs202034370
rs76666449
rs59409453
rs35640778
rs16978028
rs11117354
rs1985369
rs9398196
rs6536702
rs3785074
rs10805346
rs80324517
rs11412296
rs7705526
rs35446936
rs73730598
rs6776756
rs10768683
rs871134
rs7099229
rs73581419
rs2056726
rs143190905
rs10905255
rs4498805
rs182059586
rs131797
rs2282764
rs10112752
rs10840270
rs117630647
rs7790856
rs141214782
rs8105767
rs11212631
rs2230590
rs2967355
rs6669563
rs1003322
rs932002
rs56799554
rs17445108
rs3891167
rs7772289
rs939916
rs145114957
rs137901416
rs7164950
rs9955360
rs77231040
rs61748181
rs12451892
rs41269079
rs111950327
rs10845387
rs11991877
rs142426306
rs6881568
rs78491606
rs3093888
rs1957937
rs2538745
rs2293579
rs185174247
rs1332941
rs1907702
rs76219171
rs12412214
rs10774624
rs117034449
rs112394943
rs6007020
rs5742915
rs45604339
All − MR Egger
All − Inverse variance weighted
−2
−1
0
1
2
MR effect size for
'telomere length || id:ieu−b−4879' on 'IDP_dMRI_TBSS_ISOVF_Superior_fronto−occipital_fas
